# Anti-inflammatory genes in PBMCs post-spontaneous intracerebral hemorrhage

**DOI:** 10.1515/tnsci-2021-0003

**Published:** 2021-01-27

**Authors:** Doan Nguyen, Vi Tran, Alireza Shirazian, Cruz Velasco-Gonzalez, Ifeanyi Iwuchukwu

**Affiliations:** Institute for Translational Research, Ochsner Medical Center, Ochsner Health, 1514 Jefferson Highway, New Orleans, LA 70121, United States of America; Center for Outcomes and Health Services Research, Ochsner Health, 1514 Jefferson Highway, New Orleans, LA 70121, United States of America; Neurocritical Care and Neurology, University of Queensland, Ochsner Clinical School, Ochsner Medical Center, Ochsner Health, 1514 Jefferson Highway, New Orleans, LA 70121, United States of America; Neuroscience Center of Excellence, Louisiana State University Health New Orleans, School of Medicine, 2020 Gravier Street, 8th Floor, New Orleans, LA 70112, United States of America; Department of Neurosurgery, Louisiana State University Health New Orleans, School of Medicine, 2020 Gravier Street, 7th Floor, New Orleans, LA 70112, United States of America

**Keywords:** intracerebral hemorrhage, gene expression, peripheral blood mononuclear cells, clinical outcome

## Abstract

**Background:**

Neuroinflammation is important in the pathophysiology of spontaneous intracerebral hemorrhage (ICH) and peripheral inflammatory cells play a role in the clinical evolution and outcome.

**Methodology:**

Blood samples from ICH patients (*n* = 20) were collected at admission for 5 consecutive days for peripheral blood mononuclear cells (PBMCs). Frozen PBMCs were used for real-time PCR using Taqman probes (NFKB1, SOD1, PPARG, IL10, NFE2L2, and REL) and normalized to GAPDH. Data on hospital length of stay and modified Rankin score (MRS) were collected with 90-day MRS ≤ 3 as favorable outcome. Statistical analysis of clinical characteristics to temporal gene expression from early to delayed timepoints was compared for MRS groups (favorable vs unfavorable) and hematoma volume.

**Principle findings and results:**

IL10, SOD1, and REL expression were significantly higher at delayed timepoints in PBMCs of ICH patients with favorable outcome. PPARG and REL increased between timepoints in patients with favorable outcome. NFKB1 expression was not sustained, but significantly decreased from higher levels at early onset in patients with unfavorable outcome. IL10 expression showed a negative correlation in patients with high hematoma volume (>30 mL).

**Conclusions and significance:**

Anti-inflammatory, pro-survival regulators were highly expressed at delayed time points in ICH patients with a favorable outcome, and IL10 expression showed a negative correlation to high hematoma volume.

## 1 Introduction

Intracerebral hemorrhage (ICH) accounts for 15–20% of all patients with ‘stroke’ and carries a significant morbidity and mortality of greater than 60% [1]. Tremendous efforts geared towards treating the primary mechanical injury, which causes rapid disruption of the normal anatomy and physiology, have been unsuccessful in large clinical trials [1,2,3]. Hence, there is a renewed interest in the pathophysiology of secondary brain injury that leads to massive tissue damage, breakdown of the blood–brain barrier (BBB), cerebral edema, and further neuronal death [2,3,4,5].

Preclinical ICH studies have demonstrated that genes regulating oxidative and inflammatory pathways are upregulated following ICH. For instance, nuclear factor-kappa B (NF-κB) is a ubiquitous transcription factor that propagates pro-inflammatory pathways and is rapidly activated by the release of hemoglobin and peaks early in the ICH brain [6,7,8]. Using pharmacological modulators and gene knock out techniques, activation of anti-inflammatory and antioxidant genes such as peroxisome proliferation-activated receptor γ (PPARG), superoxide dismutase 1 (SOD1), nuclear factor erythroid-derived 2-like 2 (NFE2L2), also known as nuclear factor erythroid 2 (NF-E2)-related factor 2 (NRF2), and interleukin 10 (IL10) was associated with outcomes in animal ICH studies [9,10,11,12].

In our preliminary microarray study using Paxgene-collected peripheral whole blood, we identified several differentially expressed microRNA, two of which were miR-92a-3p and miR-92b-3p. Using MirWalk 2.0 and the miRTarbase database of validated gene targets, we identified mRNA for IL-10 and PPARG, and NF-κB regulated genes, as putative targets of miR-92a-3p and miR-92b-3p as similarly reported in the literature [13,14]. RNAseq analysis of peripheral whole blood collected within 48 h identified significant decreased expression of PPARG and IL10 in ICH patients with favorable outcome vs unfavorable outcome. Given that secondary brain injury and delayed development of ICH-related neurological deficits have a major impact on outcome, and based on their close association in ICH pathophysiology and neuroprotection, we postulate that temporal changes in their expression profile may be indicative of favorable or unfavorable outcome in ICH patients [4,6,15,16,17].

Peripheral blood mononuclear cells (PBMCs) constitute a major part of the systemic inflammatory cellular response to ICH [10,18,19,20,21,22,23]. Clinical and preclinical studies have implicated monocytes and lymphocytes (PBMCs) in ICH pathobiology and a suggested relationship between peripheral counts and outcomes [21,24]. However, it remains unclear if the gene expression and phenotypic patterns of circulating PBMCs contribute to the biologic mechanisms and ICH outcomes [25].

Since PBMCs and genes regulating oxidative and inflammatory pathways are integral in ICH pathology, we hypothesized that expression of these genes in circulating PBMCs will differ between ICH patients with favorable and unfavorable outcomes at early onset (within 48 h) and delayed. In this study, we examine the relationship of temporal gene expressions of NFKB1, PPARG, SOD1, NFE2L2 (NRF2), REL, and IL10 in PBMCs from ICH patients with favorable and unfavorable outcomes.

## 2 Materials and methods

### 2.1 Patient selection, sample collections, and outcome measures

Patients who were admitted to the Neurointensive Care Unit for management of acute spontaneous ICH confirmed on computed tomography (CT) scan were screened and consented. Demographic and clinical information were collected as shown in Table 1a and b.

**Table 1:** Association of patient characteristics (a) ICH volume at 24 h groups; (b) modified Rankin scale groups

(a) ICH volume (mL)					
	All	<30 mL	≥30 mL	*p* value^a^	OR/mean diff^b^ (95% CI)
	*N* = 20	*N* = 12	*N* = 8		
Race white, *N* (%)	15 (75)	9 (75)	6 (75)	0.9671	1.04 (0.13, 8.15)
Gender male, *N* (%)	13 (65)	7 (58.33)	6 (75)	0.5133	1.90 (0.27, 13.1)
Age, mean (SD, min, max)	64 (10.03, 49, 79)	67.5 (8.7, 53, 79)	58.7 (10.1, 49, 75)	0.0533	−8.75 (−17.6, 0.1)
BMI, mean (SD, min, max)	30.6 (6.19, 20.3, 46.6)	31.5 (7.11, 20.6, 46.6)	29.3 (4.6, 20.3, 35)	0.4109	−2.20 (−7.7, 3.3)
Hypertension, *N* (%)	18 (90)	11 (91.67)	7 (87.5)	0.7747	0.65 (0.03, 12.1)
DM, *N* (%)	9 (45)	6 (50)	3 (37.5)	0.6249	0.63 (0.10, 3.89)
CAD, *N* (%)	5 (25)	3 (25)	2 (25)	0.9671	1.04 (0.13, 8.15)
Admission WBC, mean (SD, min, max)	11.2 (4.1, 3.3, 22.0)	10.6 (3.15, 5.4, 14.9)	12.1 (5.50, 3.3, 22.0)	0.4850	1.55 (−3.22, 6.33)
Admission Neutrophils, mean (SD, min, max)	8.8 (4.21, 1.9, 19.8)	8.0 (3.51, 3.0, 13.2)	10.0 (5.10, 1.9, 19.8)	0.3426	2.05 (−2.48, 6.58)
ICH Location Deep Nuclei, *N* (%)	11 (55)	5 (41.67)	6 (75)	0.1962	3.54 (0.52, 24.1)
Hemicraniectomy, *N* (%)	4 (20)	3 (25)	1 (12.5)	0.6112	0.54 (0.05, 5.72)
Hematoma evacuation, *N* (%)	3 (15)	3 (25)	0	0.3113	0.16 (0.005, 5.56)

(b) Modified Rankin Scale (MRS)					
	All	0–3	4–6	*p* value^a^	OR/mean diff^b^ (95% CI)
	*N* = 20	*N* = 8	*N* = 12		
Race white, *N* (%)	15 (75)	8 (72.73)	7 (77.78)	0.8398	0.81 (0.10, 6.26)
Gender male, *N* (%)	13 (65)	9 (81.82)	4 (44.44)	0.1279	4.64 (0.64, 33.54)
Age, mean (SD, min, max)	64 (10.03, 49, 79)	60.5 (10.41, 49, 78)	68.2 (8.19, 55, 79)	0.0888	−7.6 (−16.4, 1.06)
BMI, mean (SD, min, max)	30.6 (6.19, 20.3, 46.6)	29.6 (6.16, 20.3, 40.9)	31.9 (6.37, 25.8, 46.6)	0.4345	−2.2 (−8.2, 3.7)
Hypertension, *N* (%)	18 (90)	10 (90.91)	8 (88.89)	0.8874	1.23 (0.06, 22.9)
DM, *N* (%)	9 (45)	4 (36.36)	5 (55.56)	0.4367	0.49 (0.08, 2.94)
CAD, *N* (%)	5 (25)	2 (18.18)	3 (33.33)	0.4946	0.48 (0.06, 3.81)
Admission WBC, mean (SD, min, max)	11.2 (4.18, 3.3,‘22.0)	10.8 (3.89, 3.34, 15.1)	11.8 (4.69, 6.21, 22.0)	0.6128	0.98 (−5.1, 3.1)
Admission Neutrophils, mean (SD, min, max)	8.8 (4.21, 1.9, 19.8)	8.2 (3.7, 1.9, 13.2)	9.6 (4.88, 3.5, 19.8)	0.4776	(−1.39 (−5.61, 2.82)
ICH Location Deep Nuclei, *N* (%)	11 (55)	7 (63.64)	4 (44.44)	0.4367	2.0 (0.33, 12.2)
Hemicraniectomy, *N* (%)	4 (20)	2 (18.18)	2 (22.22)	0.8327	0.78 (0.08, 7.08)
Hematoma evacuation, *N* (%)	3 (15)	1 (9.09)	2 (22.22)	0.5114	0.42 (0.03, 5.3)
Time to consent, HR, median (IQR)	28.88 (20.05)	30.17 (23.42)	16.45 (21.597)	0.0193	

BMI – body mass index; DM – diabetes mellitus; CAD – coronary artery disease; ICH – intracerebral hemorrhage; SD – standard deviation; WBC – white blood cell count.

a for Fisher’s exact test or *t*-test with Satterthwaite correction.

b OR from logistic regression with Firth correction for ICH ≥ 30 and, for example, White vs Black; Mean Difference (ICH > 30 – ICH < 30).

Venous blood samples (5 mL) were collected in sodium citrate tube within 48 h of presentation. Venous blood samples and PBMCs were categorized as “early” if collected within 48 h of symptom onset and “delayed” if collected greater than 72 h from symptom onset. The median time of blood collection for early onset was 28.88 h (Table 1). PBMCs were prepared from whole blood per manufacturer’s protocol using the Lymphoprep Ficoll and Sepmate Tube (StemCell Technologies, Vancouver, CN). PBMC counts were determined using a TC20 cell counter (Bio-Rad, Hercules, CA) and prepared for freezing in RMPI media (ThermoFisher Scientific, Waltham, MA) with 10% heat-inactivated fetal bovine serum (FBS, Atlanta Biologicals) and 10% DMSO (Corning Inc., Corning, NY) using standard protocol for freezing live cells. Samples were stored in liquid nitrogen until use.

Data on demographics, clinical, radiological characteristics, and hospital length of stay were collected. Patients were contacted via a telephone call at 90 days from clinical presentation to determine a modified Rankin score (MRS) using a standard questionnaire. We defined favorable outcome as 90-day MRS ≤3 and unfavorable outcome as 90-day MRS 4–6.


**Informed consent:** Informed consent has been obtained from all individuals included in this study.
**Ethical approval:** The research related to human use has been complied with all the relevant national regulations, institutional policies, and in accordance with the tenets of the Helsinki Declaration and has been approved by the authors’ institutional review board or equivalent committee.

### 2.2 Total RNA extraction and real-time RT-PCR analysis

Frozen PBMCs were rapidly thawed in a 37°C water bath with gentle agitation for 1–2 min, transferred to a 15 mL centrifuge tube containing 5 mL of RMPI media +10% FBS, and centrifuged at 400 × *g* at room temperature for 10 min. The pellet was quickly prepared for total RNA extraction using the miRNeasy microRNA kit per manufacturer’s protocol (Qiagen, Valencia, CA). Briefly, the PBMC pellet was lysed in Qialysis buffer and phase-separated using chloroform, transferred to a column, washed, and resuspended in 14 µL RNase-free water. RNA concentration was determined using the NanoDrop spectrophotometer (Thermo Fisher Scientifics, Waltham, MA). Total RNA (100 ng) was used for cDNA synthesis using the iScript cDNA Synthesis Kit (Bio-Rad). The cDNA was diluted in sterile water and 10 ng was used for real-time PCR using Taqman probe (Thermo Fisher Scientifics). The target genes include PPARG, IL10, NFKB1 (P105/p55), REL (c-Rel), SOD1, and NFE2L2. PCR amplification was carried out in a Applied Biosystem 7,500 Fast Thermal Cycler (Thermo Fisher Scientifics) in triplicate using recommended parameters for 40 cycles. Cycle threshold (Ct) data were normalized to the GAPDH control gene and relative fold change was determined using the 2^−ΔΔCt^ method. Differential gene expression was determined based on a 1.5-fold cutoff.

### 2.3 Statistical methods

Demographic and clinical characteristics were compared between ICH volume groups (<30 vs ≥30 mL) by means of a Fisher exact test (corresponding OR were obtained from a logistic regression model with Firth correction) or a *t*-test with Satterthwaite correction. Similar analyses were carried out to compare these characteristics between favorable and unfavorable outcome groups (MRS 0–3 and 4–6). Potential covariation of ICH volume at 24 h and Ct values (normalized to GAPDH) for each gene (IL10, NFKB1, NFE2L2, PPARG, SOD1, and REL) at 0–2 days – and separately at 3–5 days – was estimated by means of raw Spearman correlations. Covariation of ICH volume at 24 h and fold change by the 2^−ΔΔCt^ method from 0–2 days to 3–5 days was also assessed by raw Spearman correlations. *P*-values are considered as indicative of potential associations. Gene expression was estimated using mean normalized Ct values for clinical outcome and for early (0–2 days) to delayed (3–5 days) and analyzed by the Holm-Sidak *t*-test with alpha = 0.05 in Prism 8.0 (Graphpad, San Diego, CA).

## 3 Results

### 3.1 Patient characteristics

A total of 20 consecutive patients with spontaneous ICH between November 2017 and January 2019 who met the inclusion criteria were recruited for the study. Overall, 13 were male (65%) and 90% had a prehospital diagnosis of hypertension; 9 patients (45%) had a favorable outcome (MRS 0–3). Table 1a and b describe patients’ characteristics based on ICH volume at 24 h and the comparison between patients with favorable and unfavorable outcomes based on Modified Rankin Scale. A lower ICH score at admission (≤2) was more likely in patients with favorable outcome (75 vs 8.3%). Also, there was a tendency towards younger age in the favorable outcome group and in ICH volume <30 mL group. The median time from onset to blood collection was 28.88 and the interquartile range (IQR) 20.05 h. When subset based on favorable and unfavorable outcome, the median time was 28.35 vs 15.17 h and was significantly different for favorable vs unfavorable, respectively. When the early onset (first 48 h) was subset into <12 and >12 h for the two outcome groups (MRS 0–3 vs MRS 4–6), the difference between the two groups was not significant.

### 3.2 Differential expression of mRNA in PBMCs and outcome

**Figure 1:**
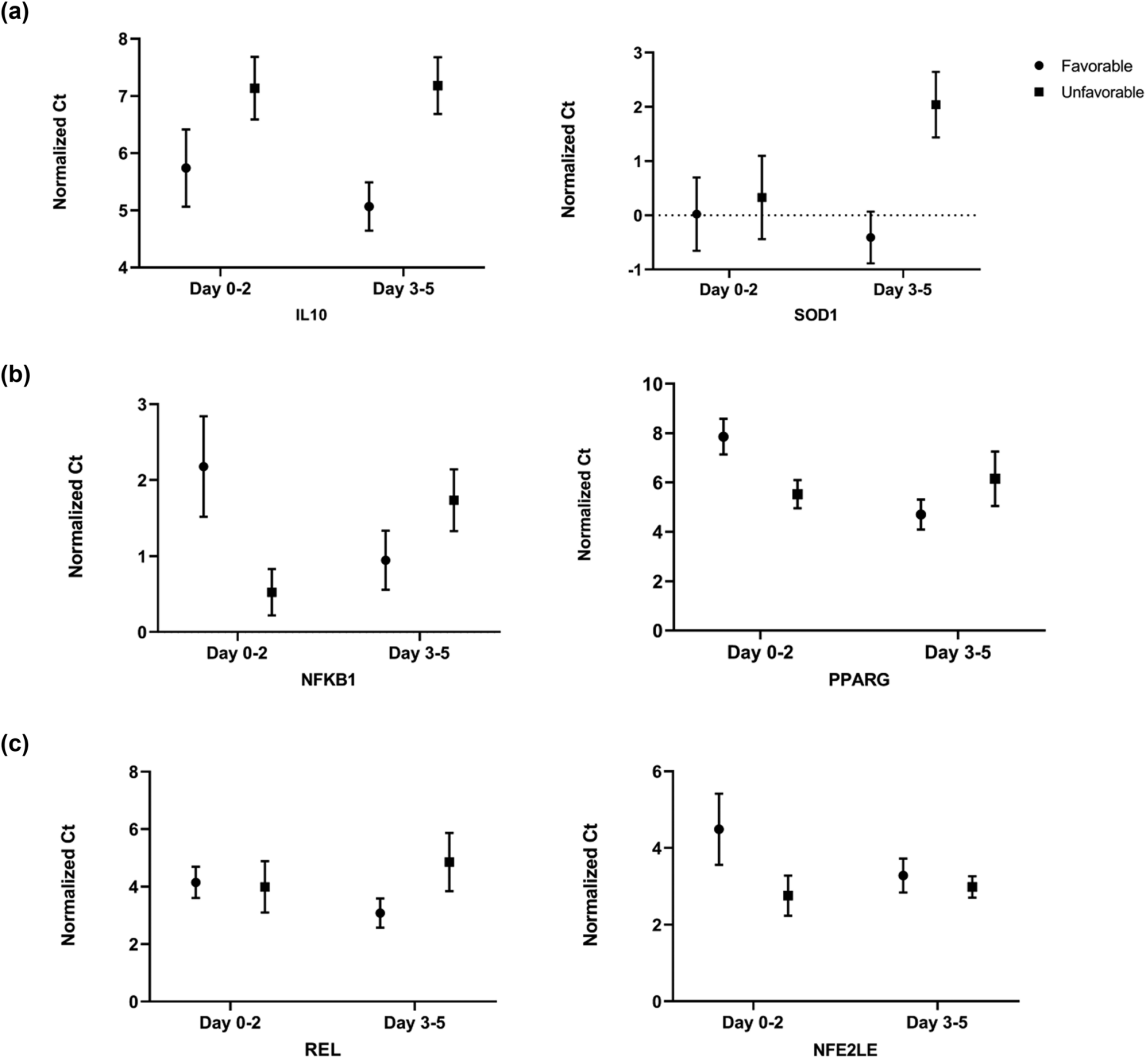
Expression of IL10, SOD1, NFKB1, REL, and PPARG in PBMCs at Day 0–2 and Day 3–5 of ICH patients with unfavorable outcome (*n* = 7) relative to ICH patients with favorable outcome (*n* = 10). A smaller normalized Ct value indicates a higher gene expression level. (a) Expressions of IL10 and SOD1 were significantly higher at Day 3–5 in favorable outcome than unfavorable outcome (*p*-value < 0.05). (b) Expressions of NFKB1 (*p* = 0.046) and PPARG (*p* = 0.027) at Day 0–2 were significant. Expressions of NFKB1 and PPARG were higher at Day 0–2 in ICH patients with unfavorable outcome. REL expression was significantly higher at Day 3–5 in favorable outcome compared to unfavorable outcome (*p* = 0.032). Expression of NFE2LE was not significant at either time points. Data are expressed as mean ± standard error of mean (SEM).

Several genes were found statistically significant in PBMCs of patient with favorable outcome vs unfavorable outcome. The early onset (first 48 h) and delayed (day 3–5) gene expression patterns for favorable and unfavorable outcomes are shown (Figure 1). Expression of IL10 at the delayed time points was statistically significant (*p*-value < 0.05) and 3-fold higher for patients with favorable compared with unfavorable outcome. In patients with favorable outcomes, the expression of IL10 increased 1.5-fold at the delayed time points over the level of the early time points, whereas in patients with unfavorable outcome, the levels remained constant from the early to delayed time points. Expression of SOD1 at the delayed time points was statistically significant (*p*-value < 0.05) and 5-folds higher in favorable than unfavorable outcomes. SOD1 expression in unfavorable outcome decreased 3.2-fold from early to delayed time points, whereas in favorable outcome, a gradual increase was seen over time. Expression of IL10 and SOD1 were not different between groups at early time points.

At the early time points, expression of NFKB1 and PPARG were significant (*p* < 0.05) and exhibited a 2.3-fold and 5-fold increase, respectively, in unfavorable outcome than favorable outcome. No significant difference was seen at the delayed time points. For NFKB1 in unfavorable outcome, expression of NFKB1 significantly decreased 2.3-fold from the early time point over the delayed time points (*p* < 0.05). The opposite pattern of 2.3-fold increase from the delayed time points over the early time points for patients with favorable outcome is noteworthy. In unfavorable outcome, PPARG expression decreased slightly at 1.5-fold from the early to the delayed time points, in contrast to favorable outcome in which PPARG expression significantly increased greater than 9-fold (*p* < 0.05).

Another member of the NF-κB family of transcription factors, REL expression at the early time points was not different for patients with favorable and unfavorable outcome. However, at the delayed time point, REL expression was statistically different (*p* < 0.05). Expression of REL increased 2.9-fold from the early to the delayed timepoints in patients with favorable outcome (*p* < 0.05), while in patients with unfavorable outcome, REL expression did not change. Though NFE2L2 was 2-fold higher in unfavorable outcome compared to favorable outcome, NFE2L2 expression at the delayed time point was similar between outcome groups. No change in NFE2L2 was seen for unfavorable outcome; however, in patients with favorable outcome, NFE2L2 expression increased 3.2-fold at delayed time point.

In general, at the early time point the expression of NFKB1, PPARG, and NFE2L2 in unfavorable outcome was higher than that in favorable outcome, while at the delayed time point expression of IL10, SOD1, and REL was higher in patients with favorable outcome. Interestingly, the observed higher early expression of PPARG in unfavorable outcome was reversed at the delayed time point, with a higher expression in the favorable outcome group. In summary, gene expression increased from the early time points to the delayed time points in patients with favorable outcome.

### 3.3 Correlation of PBMCs gene expression, ICH volume at 24 h, and clinical/demographic characteristics

**Figure 2:**
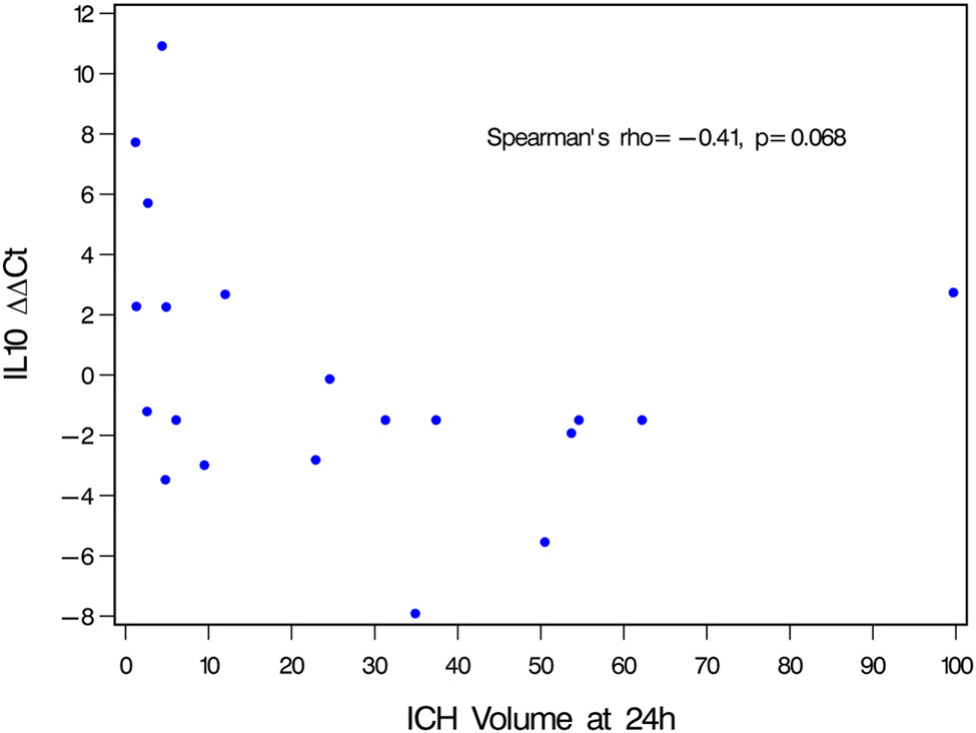
Plot of ICH volume at 24 h vs IL10 fold change. Expression of IL10 showed a negative correlation to high hematoma volume (>30 mL).

It is known that ICH volume is an independent predictor of outcome. We analyzed the effect of each gene expression with known covariates of ICH outcome such as age, gender, ICH location, and diabetes mellitus (Table 2, Suppl Table S1). The table shows unadjusted and partialled Spearman correlation of genes and ICH volume at 24 h. Unadjusted analysis showed a potential direct association between ICH volume and each of IL10 and SOD1 (rho = 0.41, *p* = 0.073).The association for ICH volume and IL10 expression increases slightly from 0.4091 (*p* = 0.0732) to 0.4801 (*p* = 0.0375) and becomes significant when adjusted for age, which suggests the strength of association becomes more likely for IL10 expression and ICH volume (Table 2). The strength of the association is stronger with ICH location (rho = 0.4666, *p* = 0.0440) and surgical intervention (rho = 0.5587, *p* = 0.0129; rho = 0.5339, *p* = 0.0185). When ICH volume was correlated to the temporal fold change from the early day 0–2 time points to delayed 3–5 time points for the individual genes, only IL10 expression showed a significant and negative correlation (Table 3). The inverse correlation indicates that larger values of ICH volume at 24 h tend to associate with smaller fold change of IL10 from early 0–2 days to delayed 3–5 days. Although IL10 was not found statistically significant (*p* = 0.068), its expression and direction of change suggest increasing ICH volume has a negative impact on IL10 expression (Figure 2). Interestingly, the association between ICH volume and SOD1 expression increases slightly when adjusting for diabetes mellitus, but remains unchanged when adjusted for every other covariates. Lastly, REL expression showed a significant (*p* = 0.042) and moderate association when adjusted for hemicraniectomy. Due to the small sample size, a multivariate analysis was not done to identify the effect of the various covariates on gene expressions.

**Table 2:** Correlation of gene expression and ICH volume at 24 h and clinical/demographic status

	IL10		NFKB1		NFE2L2		PPARG		SOD1		REL	
	rho**	*p* value	rho	*p* value	rho	*p* value	rho	*p* value	rho	*p* value	rho	*p* value
**Unadjusted**	0.4091	0.0732	0.206	0.3835	0.2526	0.2826	0.0451	0.85	0.409	0.0733	0.294	0.354
**Partialled for** Age	0.4801*	0.0375	0.2405	0.3211	0.2256	0.353	0.0264	0.9148	0.3901	0.0987	0.499	0.141
ICH location deep nuclei	0.466*	0.044	0.2379	0.3267	0.2552	0.2915	0.0385	0.8754	0.4088	0.0822	0.38	0.279
Hemicraniectomy	0.558*	0.0129	0.2651	0.2726	0.2858	0.2354	0.0963	0.6947	0.417	0.0757	0.648*	0.042
Hematoma evacuation	0.534*	0.0185	0.2359	0.3307	0.2389	0.3246	0.0669	0.7855	0.3715	0.1173	0.572	0.084

*p < 0.05; **Spearman’s correlation coefficients.

**Table 3:** Spearman correlations between ICH Volume at 24 h and fold change (2^−ΔΔCt^) from 0–2 to 3–5 days

	rho	*p* value
IL10	−0.4511	0.0459*
NFKB1	−0.1729	0.4659
NFE2L2	−0.2286	0.3324
PPARG	−0.1368	0.5651
SOD1	−0.3158	0.1750
REL	0.2273	0.5015

*p < 0.05.

## 4 Discussion

The present study identified that IL10 expression was negatively affected in patients with high ICH volume, which is an independent predictor of outcome. The study also showed that in patients with favorable outcome, an increasing trend in gene expression for PPARG, IL10, REL, NFKB1, and NFE2L2 from the early onset (0–48 h) to the delayed (within 72–120 h) is associated with favorable outcome.

Our results from human patients with ICH suggest that the role of regulators of anti-inflammatory and protective factors based on PBMC gene expression is important in predicting ICH outcomes. An important observation from our results is the overall higher expression of these transcriptional regulators, namely PPARG, REL, and NFE2L2 in PBMC at a delayed time point (>72 h), which falls within the time point of progressive cerebral edema after ICH [26,27]. Our findings are in-line with those reported by Aronowski’s group and others on the anti-inflammatory and synergistic effects of PPARγ, along with NRF2 and SOD1, to affect NF-κβ1 pro-inflammatory function [11,12,15,20]. This shift in gene expression is observed in the increased expression of REL at a delayed time point in ICH patients with favorable outcome and suggests a shift from pro-inflammatory to activation of pro-survival mediators of the Rel/NF-κB subtype [17]; hence, the hypothesis that an increased expression of anti-inflammatory genes reduces post-ICH complications and is associated with favorable outcome. Alternatively, the observed increased expression of anti-inflammatory genes is a compensatory response to increased expression of the pro-inflammatory NF-κβ1 as observed among the patients with favorable outcome at the delayed time point.

In our study, NFKB1 expression in ICH patients with favorable outcome was increased and remained higher from the early onset day 0–2 to delayed day 3–5. Conversely, in ICH patients with poor outcome, expression of NFKB1 decreased from the early onset to delayed time points. This suggests that the early activation of NF-κβ1 is important. The NF-κβ family of transcription factors is involved in many aspects of immune responses as well as in cell survival. NF-κβ activation is mediated by both canonical and noncanonical signaling pathways which, upon induction, are translocated to the nucleus and form specific NF-κβ complexes with other Rel/NF-κβ subtypes to affect gene transcription [28]. Although we did not directly measure NF-κβ activation, activation of the canonical pathway is mostly associated with NF-κβ1 p50/RelA and p50/c-Rel dimer complexes [17,28]. The increase in REL expression in patients with favorable outcome suggests the induction of NF-κβ1 response and activation of c-Rel mediated transcription of target genes involved in survival and protective response.

Along this line of anti-inflammatory, protective effect of increased expression of IL10, we found a significant change in the expression of other protective activators, PPARG and REL, at delayed day 3–5. SOD1, a superoxide dismutase enzyme abundant in all cells function to break down superoxide radicals in order to prevent radicals-induced toxicity. In the unfavorable outcome group, the expression of SOD1 increased at early onset, but was not sustained at delayed day 3–5. This suggests that sustained SOD1 expression is needed as part of the anti-inflammatory, protective effect following acute ICH [29,30].

Monocytes and lymphocytes infiltrate the injured brain early (within 6–12 h), peaking by 48 h and play an important role in ICH-related inflammation [18,19,24]. One study reported the ratio of CD4+/CD8+ T-lymphocytes corresponded to outcomes [24]. Other clinical studies have reported findings of the relationship of peripheral monocyte heterogeneity with outcomes in ICH patients [21,22,23]. Recruited monocytes to the injured brain transform into macrophages and release pro-inflammatory cytokines and chemokines causing BBB damage, hematoma expansion, cerebral edema, and further neuronal cell death [10,25]. Expectedly, our study suggests that, based on IL10 gene expression pattern following ICH, the M2-monocyte/macrophage subtype may be elevated and associated with favorable outcomes [31,32]. Furthermore, increased expression of the transcriptional regulator PPARG with anti-inflammatory properties may be involved in mediating the shift to the M2 phenotype [33]. Further human studies on the heterogeneity of peripheral inflammatory cells, cell clustering, and their relationship with ICH outcomes are needed.

### 4.1 Study strengths and limitations

It is recognized that ICH volume is an independent predictor of clinical outcome after spontaneous ICH. Our study suggests that expression levels of IL10, an anti-inflammatory, M2 monocytes-associated cytokine, correlate to ICH volume and outcome. The result from the study also showed that measuring pattern of gene expression (PPARG, REL, IL10, NFE2L2) in PBMCs at early onset and 3–5 days post-admission may provide indication for clinical intervention and to predict outcome. Our small sample size limits the ability to account for variations in gene expression influenced by age, sex, race, and premorbid conditions. Although we looked at temporal gene expression changes at early onset and delayed, our sampling method of combining and grouping PBMC samples as day 0–2 and day 3–5 may also account for gene expression variations and may not provide a clear, temporal changes from day 0 to day 5. Even though we did not see a statistically significant difference between the two groups, time as a continuous variable should be considered when evaluating the fluctuation of gene expression of PBMCs during the first 48 h. Also, our study did not take into account the gene-gene interaction effects.

## 5 Summary

In this study, we have reported the relative differences in expression of a set of transcriptional regulators and pro- and anti-inflammatory factors in PBMCs of ICH patients with favorable and unfavorable outcomes. As these cells play an important role in secondary brain injuries including inflammation that may influence ICH outcome, PBMCs expression of these regulators and genes from early to delayed may indicate favorable or poor recovery. Specifically, we identified in ICH patients with favorable outcome that expression of PPARG, REL, NFE2L2, NFKB1, and IL10 in PBMCs showed an increasing trend from early to delay time points, whereas in ICH patients with unfavorable outcome, expression of these genes remained unchanged. We found that IL10 expression is negatively correlated to ICH volume, an independent predictor of outcome. These results suggest that increased expression of these regulators in PBMCs promotes and activates anti-inflammatory genes to promote favorable outcome post-ICH.

## Abbreviations

BBB: blood—brain barrier
IL10: interleukin-10
SOD1: superoxide dismutase 1
NFE2L2: nuclear factor of erythrocyte-2-related factor 2
NRF2: NF-E2-related factor 2
PPARG: peroxisome proliferation-activated receptor gamma
GAPDH: glyceraldehyde dehydrogenase
COX-2: cyclooxygenase-2
CSF: cerebrospinal fluid
Ct: cycle threshold
CT: computed tomography
ICH: intracerebral hemorrhage
miRNA: microRNA
NFKB1: NF-κB, nuclear factor-kappa beta-1
REL: REL proto-oncogene
NFKB: subunit
